# Change of Retinal Vessel Density After Lowering Intraocular Pressure in Ocular Hypertension

**DOI:** 10.3389/fmed.2021.730327

**Published:** 2021-12-09

**Authors:** Xuhao Chen, Ying Hong, Haohao Di, Qianru Wu, Di Zhang, Chun Zhang

**Affiliations:** ^1^Department of Ophthalmology, Peking University Third Hospital, Beijing, China; ^2^Beijing Key Laboratory of Restoration of Damaged Ocular Nerve, Peking University Third Hospital, Beijing, China; ^3^Department of Ophthalmology, Zhengzhou Second Hospital, Zhengzhou, China

**Keywords:** ocular hypertension, optical coherence tomography angiography, vessel density, intraocular pressure, latanoprost

## Abstract

**Purpose:** To investigate the relationship between retinal microvasculature changes and intraocular pressure (IOP) for ocular hypertension (OHT) patients and further assess the factors associated with retinal microcirculation changes.

**Methods:** This was a single-center prospective study designed for OHT patients, which consisted of two visits. After collecting baseline data of those who met the eligibility criteria, these patients were treated with latanoprost 0.005% ophthalmic solution for 4 weeks. Peripapillary vessel density (VD) of radial peripapillary capillaries (RPC) layer, macular VD in both superficial and deep layers, and foveal avascular zone (FAZ) area were measured by optical coherence tomography angiography (OCTA) before and after the treatment. We compared the changes in IOP and VD among the two visits by paired-sample *t*-test. Bonferroni correction was applied. Factors associated with VD changes were analyzed by linear regression analysis.

**Results:** Thirty-four eyes of thirty-four patients were included. The mean IOP decreased by 6.5 ± 2.2 mmHg (*p* < 0.001). The peripapillary RPC VD increased significantly from 51.8 ± 2.5 to 53.0 ± 3.1% (Adjusted-*p* = 0.012). We found no significant difference in detailed sectors of the peripapillary region after correction. In the macular area, both the superficial and deep layers in foveal (superficial: 0.2 ± 1.9%, *p* = 0.523; deep: 0.0 ± 2.3%, *p* = 0.969) and parafoveal (superficial: 0.3 ± 3.0%, *p* = 0.565; deep: 0.5 ± 3.1%, *p* = 0.423) VD remained unchanged. The decrease of the mean FAZ area was insignificant (*p* = 0.295). The percentage of IOP reduction (β = 0.330, *p* = 0.031) and the baseline RNFL thickness (β = 0.450, *p* = 0.004) significantly correlated with the percentage of peripapillary RPC VD improvement in the multivariate linear regression analysis.

**Conclusion:** The peripapillary VD in OHT patients increased after the reduction of IOP. The mild change of IOP did not alter the microcirculation in the macula. In addition, the percentage of IOP change and the baseline RNFL thickness were independent factors for the peripapillary RPC VD improvement.

## Introduction

Intraocular pressure (IOP) and retinal circulation were influenced by autoregulation in normal tissue (1, 2). A mild change of IOP within a short time exerts little influence on the peripapillary (3) or macular (3, 4) microcirculation. With the broader range of IOP changes, however, such homeostasis will be affected. Previous studies have revealed that an IOP spike is associated with reduced retinal perfusion in healthy controls (5, 6) and patients with narrow anterior chamber angles (4, 7). However, based on the models' nature, the long-term effect of IOP change on retinal circulation remained unsolved. Recent studies mainly discussed such interplay on glaucoma patients, with an emphasis on the impact of surgeries (8–13) and medication applications (14–18). Given the damaging retinal circulation with glaucoma progression (19), the correlation between the extent of microcirculation improvement and IOP reduction was inconsistent among studies. In comparison, ocular hypertension (OHT) patients present no signs of glaucomatous defects but suffer the risk of developing and progressing into primary open-angle glaucoma (POAG) with long-term IOP elevation (20–22). Previous studies revealed that the IOP reduction for OHT showed little impact on the ophthalmic artery (23). However, based on the nature of OHT, the retinal microcirculation may be influenced by topical hypotensive treatment (18). The peripapillary and macular microcirculation and their associations with IOP change for OHT were not thoroughly evaluated, which may affect the robustness to its therapeutic value. Further, the factors associated with microcirculation changes still need to be explored.

Optical coherence tomography angiography (OCTA) is an updated imaging technique based on the mechanism of optical coherence tomography (OCT) (24). It has been widely used as a non-invasive tool to assess glaucoma patients' retinal microcirculation (24, 25). The motion contrast generated by red blood cells provides a means to visualize the retinal microvasculature in both the macula and peripapillary region (25). With OCTA, the capillaries were automatically divided into different layers of both regions. To our knowledge, limiting studies had explored the retinal vascular response to IOP change by OCTA on OHT. Hereby, we used OCTA to assess the relationship between retinal microcirculation changes and IOP for OHT patients and further evaluated the factors associated with such vascular changes.

## Methods

This single-center prospective study was approved by the Institutional Review Board of Peking University Third Hospital (M2017242) and adhered to the tenets of the Declaration of Helsinki. Written informed consent was obtained from each participant before the enrollment.

### Participants

Patients with OHT were prospectively enrolled in the glaucoma clinics of Peking University Third Hospital between July 2018 and June 2021. The criteria for ocular hypertension had been mentioned in the previous guideline (26). Inclusion criteria for all subjects were: (1) age between 10 and 70 years old, (2) open anterior chamber angles on gonioscopy, (3) IOP >21 mmHg, (4) best-corrected visual acuity (BCVA) ≥20/40 and refractive error within +3.00D and −8.00D, (5) without signs of RNFL retinal nerve fiber layer (RNFL) defect on fundus examination, (6) without signs of glaucomatous visual fields defects, and (7) treatment-naïve OHT. The study excluded those with (1) an IOP decrease of <10% of baseline after the treatment, (2) a history of systemic diseases including ischemic heart disease and diabetes, (3) a history of retinal or neurological disease, ocular trauma, or surgeries, and (4) poor OCT scans with scan quality (SQ) <6, the presence of motion artifacts or segmentation errors. One eye of each subject was enrolled in the study.

The study was comprised of two visits for each patient. All participants underwent standard ophthalmic examinations, including a best-corrected visual acuity measurement, slit-lamp examination, gonioscopy, Goldmann applanation tonometry, fundus examination, central corneal thickness (CCT), visual fields, and OCTA during the first visit. After collecting baseline data, the patients were treated topically with latanoprost 0.005% ophthalmic solution (XALATAN, Pfizer Manufacturing Belgium NV, Puurs, Belgium) once for four weeks in the ocular hypertension eye. The IOP and OCTA were re-evaluated after the four-week treatment at the same time of a day as one's first visit.

### OCTA Image Acquisition and Processing

**Figure 1:**
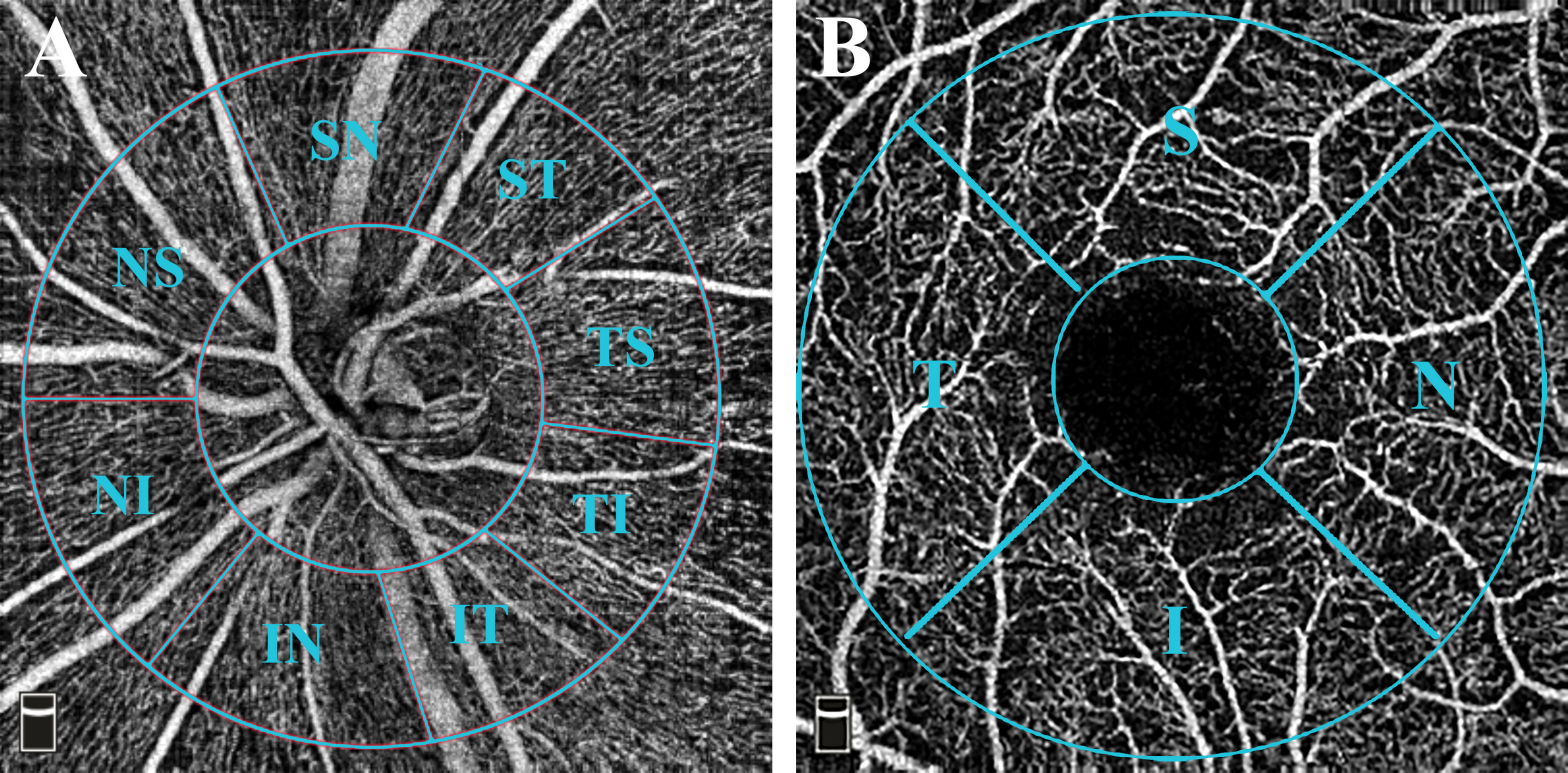
OCTA measurement of vessel density. **(A)** Angio Disc 4.5 × 4.5 mm scan (left eye). The inner-circle represented the inside disc sector. The peripapillary region is defined by a 2-mm and a 4-mm ring centered on the disc. The image presented the peripapillary sectors according to a modified Garway-Heath method. The eight sectors included Nasal Superior (NS), Nasal Inferior (NI), Inferior Nasal (IN), Inferior Temporal (IT), Temporal Inferior (TI), Temporal Superior (TS), Superior Temporal (ST), and Superior Nasal (SN) regions. **(B)** Angio Retina 3 × 3 mm scan (right eye). The 1-mm circle in the center referred to the fovea. The parafovea region was defined as a 1 mm-wide annulus extending from a 1 mm circle centered on the fovea. The central foveal and parafoveal subfields were divided in accordance with the early treatment of diabetic retinopathy study (ETDRS) retinal grid definition, which included Nasal (N), Superior (S), Temporal (T), and Inferior (I) regions.

The OCTA scans were acquired via the Avanti spectral-domain system (RTVue-XR Avanti, software version 2017.1.0.155; Optovue, Inc.; Fremont, CA, USA). The perfused retinal vasculature was identified according to a split-spectrum amplitude-decorrelation angiography algorithm by capturing the motion of particles. Trained and experienced technicians were in charge of acquiring the scans. The Angio Disc 4.5 × 4.5 mm and Angio Retina 3.0 × 3.0 mm scans were obtained for both eyes within the same visit of the participants. Under these scan patterns, B-scans were equally spaced along the horizontal and vertical dimensions. Each scan was repeated at least twice. The built-in Angiovue software was centered on the optic disc and fovea automatically after the imaging process. Vascular information was featured quantitatively as vessel density (%), which calculated the proportion of perfused blood vessels within the measured area. The peripapillary region was defined as a 1 mm-wide annulus extending from a 2 mm ring centered on the disc, based on the disc margin. A modified Garway-Heath sector grid was overlaid on the Angio Disc en face images, dividing the peripapillary region into eight sections (Figure 1A) (27). The VD was measured on the radial peripapillary capillaries (RPC) plexus ranged from the inner limiting membrane (ILM) to the lower boundary of RNFL. Similarly, the parafovea region was defined as a 1 mm-wide annulus extending from a 1 mm circle centered on the fovea. The fovea center was automatically identified by searching for the thinnest part of the retinal slab ranging from ILM to the inner plexiform layer (IPL). The central foveal and parafoveal subfields were divided in accordance with the early treatment of diabetic retinopathy study (ETDRS) retinal grid definition (Figure 1B). And the VD of both superficial and deep capillary plexus was measured on each subfield. Specifically, the superficial layer ranged from ILM to 10 μm above the IPL, while the deep layer ranged from 10 μm above IPL to 10 μm below the outer plexiform layer (OPL). A built-in Projection Artifact removal (PAR) algorithm was automatically applied to minimize the artifacts from the overlying vasculature. The foveal avascular zone (FAZ) area was automatically detected and measured. Any misalignment of OCTA scans was corrected manually. In order to repeat the scan location of the first visit, a follow-up mode was applied to both the retina and disc images. The VD will be excluded from the final analysis if one of the subfields lacks>30% of pixels as calculated by the built-in software due to scan de-centration. SQ indicated the image quality based on the signal strength index, eye motion, and focus. All scans were individually reviewed by two investigators (XC, HD) for evaluation of scan quality. The repeatability of the OCTA VD and FAZ measurement was assessed by the same investigators on different days in a random subset of ten eyes from our cohort.

### Statistical Analysis

The sample size was preliminarily estimated by PASS (Version 11.0; NCSS Statistical Software, Kaysville, UT, USA). To detect a paired difference between the two visits, a sample size of 30 can achieve more than 80% power with a significance level of 0.05. Data analysis was performed using SPSS Statistics Software (Version 24.0; IBM Corp., Armonk, NY, USA). Figures were drawn by GraphPad Prism (Version 7.0; GraphPad Software, Inc., San Diego, CA, USA). The repeatability of the OCTA measurements was assessed using the intraclass correlation coefficient (ICC) by employing a two-way random-effects model. The Shapiro-Wilk test and Q-Q plots were used to assess the normality of each variable. Data were presented as means ± standard deviation (SD). Data before and after the treatment were compared and analyzed with a paired-sample *t*-test or Wilcoxon signed-rank test based on the normality. ΔVD and ΔIOP were defined as the percentage of change between the two visits. Regression analysis was performed to investigate the factors associated with the decrease of peripapillary VD. A univariate model was first introduced to explore potential factors. Variables with *p* < 0.10 in the univariate model were then introduced to a multivariate model by the stepwise method. *P* < 0.05 was considered statistically significant. *P*-values were adjusted by Bonferroni correction considering the multiple comparisons of vessel density in scans before and after the treatment.

## Results

### Demographics

Following the eligibility criteria, a total of 34 eyes of 34 patients were included in the final analysis. Table 1 shows the demographic, clinical, and optic nerve head (ONH) structure of all the subjects. The mean age was 32.7 ± 13.0 years, ranging from 12 to 68 years. The mean RNFL thickness was 114.1 ± 12.2 μm at baseline, while the mean parafoveal retinal thickness was 328.3 ± 14.7 μm. The mean IOP before treatment was 24.7 ± 2.6 mmHg (range: 22–32 mmHg). Latanoprost decreased the IOP by 6.5 ± 2.4 mmHg (*t* = 15.612, *p* < 0.001) after four weeks. The difference between the OCTA SQ of the two visits was insignificant in both scans (Angio Disc: *Z* = −0.423, *p* = 0.672; Angio Retina: *Z* = −0.664, *p* = 0.507). The ICCs for VD and FAZ measurement ranged from 0.967 to 1.000 (all *p* < 0.001, Supplementary Table 1).

**Table 1:** Demographics of the participants (*N* = 34).

**Characteristic**	**Mean ± SD; No. (%)**
Age (years)	32.7 ± 13.0
**Gender**	
Male	19 (56)
Female	15 (44)
**Eye**	
OD	23 (68)
OS	11 (32)
SBP	120.8 ± 12.1
DBP	74.5 ± 12.0
BCVA (logMAR)	0.01 ± 0.04
SE	−3.46 ± 2.66
CCT (μm)	556.2 ± 32.0
C/D area ratio (%)	0.25 ± 0.13
C/D vertical ratio (%)	0.50 ± 0.18
C/D horizontal ratio (%)	0.45 ± 0.16
Rim area (mm^2^)	1.6 ± 0.36
Disc area (mm^2^)	2.17 ± 0.48
Cup volume (mm^3^)	0.12 ± 0.10
**IOP (mmHg)**	
Pre-treatment	24.7 ± 2.6
Post-treatment	18.2 ± 2.6
**Angio disc SQ**	
Pre-treatment	8.4 ± 0.7
Post-treatment	8.4 ± 0.8
**Angio retina SQ**	
Pre-treatment	7.7 ± 1.0
Post-treatment	8.0 ± 0.9

*BCVA, best-corrected visual acuity; CCT, central corneal thickness; C/D, cup/disc; DBP, diastolic blood pressure; DS, diopter of spherical power; IOP, intraocular pressure; SBP, systolic blood pressure; SE, spherical equivalent; SQ, scan quality*.

### Peripapillary VD

We compared RPC VD's data to evaluate the peripapillary microcirculation changes after IOP decrease for four weeks. The VD in the peripapillary region, indicating RPC's conditions, significantly increased from 51.8 ± 2.5% to 53.0 ± 3.1% (*t* = −3.664, *p* = 0.001) (Table 2). However, the VD inside the disc, which displayed the microcirculation of ONH, was not increased (0.4 ± 3.7%, *t* = −0.697, *p* = 0.504). In the comparison of a detailed segmentation, six of the eight sectors showed a significant increase. However, after the Bonferroni correction, only the overall peripapillary RPC VD increase remained statistically significant (adjusted-*p* = 0.01).

**Table 2:** Peripapillary vessel density improved after IOP decrease (*N* = 34).

**Sectors**	**Pre-treatment (%, Mean ± SD)**	**Post-treatment (%, Mean ± SD)**	***t***	***p***	**Adjusted** ***p***
Inside disc	50.6 ± 5.9	51.0 ± 6.2	−0.676	0.504	1.00
Peripapillary	51.8 ± 2.5	53.0 ± 3.1	−3.664	**0.001**	**0.01**
NS	46.5 ± 4.7	47.3 ± 5.5	−2.029	0.051	0.51
NI	45.5 ± 4.5	46.8 ± 4.8	−2.968	**0.006**	0.06
IN	50.2 ± 4.2	52.0 ± 5.0	−2.691	**0.011**	0.11
IT	57.8 ± 4.8	59.1 ± 4.2	−2.615	**0.013**	0.13
TI	54.7 ± 3.4	55.5 ± 3.4	−1.718	0.095	0.95
TS	56.3 ± 3.3	57.8 ± 3.5	−2.785	**0.009**	0.09
ST	56.7 ± 3.6	58.1 ± 3.5	−2.545	**0.016**	0.16
SN	50.6 ± 4.8	52.0 ± 4.9	−2.408	**0.022**	0.22

*The peripapillary region was divided into eight sectors including Nasal Superior (NS), Nasal Inferior (NI), Inferior Nasal (IN), Inferior Temporal (IT), Temporal Inferior (TI), Temporal Superior (TS), Superior Temporal (ST), and Superior Nasal (SN). P < 0.05 were marked in bold. P-values were adjusted by Bonferroni correction*.

### Macular VD

Both the superficial and the deep layers were investigated in the macula. In the fovea region, the vessel density remained stable after the treatment in both layers (superficial layer: 0.2 ± 1.9%, *t* = −0.646, *p* = 0.523; deep layer: 0.0 ± 2.3%, *t* = 0.039, *p* = 0.969). In a detailed analysis of FAZ, its mean area decreased from 0.331 ± 0.104 mm^2^ to 0.328 ± 0.102 mm^2^, but such a trend was insignificant (*t* = 1.066, *p* = 0.295). Similarly, the changes in parafoveal region were insignificant after the IOP decreased (superficial layer: 0.3 ± 3.0%, *t* = −0.583, *p* = 0.565; deep layer: 0.5 ± 3.1%, *t* = 0.813, *p* = 0.423). The detailed segmentation of the parafoveal region revealed no significant VD increase in all the quadrants (Table 3). To analyze the potential differences in response to IOP, we further compare the VD change between the superficial and deep layers. No significant difference was found in both foveal (*t* = 0.620, *p* = 0.540) and parafoveal (*t* = −0.313, *p* = 0.756) regions.

**Table 3:** Vessel density changes in macula scan after IOP decrease (*N* = 31).

**Sectors**	**Superficial layer**				**Deep layer**			
	**Pre-treatment**	**Post-treatment**	***t***	***p***	**Pre-treatment**	**Post-treatment**	***t***	***p***
	**(%, Mean ± SD)**	**(%, Mean ± SD)**			**(%, Mean ± SD)**	**(%, Mean ± SD)**		
Fovea	16.2 ± 6.4	16.4 ± 5.8	−0.646	0.523	30.4 ± 6.8	30.4 ± 6.1	−0.039	0.969
Parafovea	50.0 ± 2.9	50.3 ± 2.9	−0.583	0.565	52.9 ± 3.1	53.5 ± 3.1	−0.813	0.423
Temporal	48.7 ± 3.0	48.8 ± 2.9	−0.338	0.738	53.2 ± 2.8	53.2 ± 3.0	−0.017	0.987
Superior	51.0 ± 3.5	51.7 ± 2.8	−1.086	0.286	52.6 ± 3.9	53.0 ± 3.7	−0.556	0.582
Nasal	49.5 ± 2.8	49.4 ± 3.5	0.143	0.887	53.6 ± 3.6	54.2 ± 3.6	−0.984	0.333
Inferior	50.9 ± 3.9	51.3 ± 3.8	−0.710	0.484	52.1 ± 3.4	52.9 ± 3.4	−1.079	0.289

*P < 0.05 were considered statistically significant*.

### Factors Associated With RPC VD Increment

**Figure 2:**
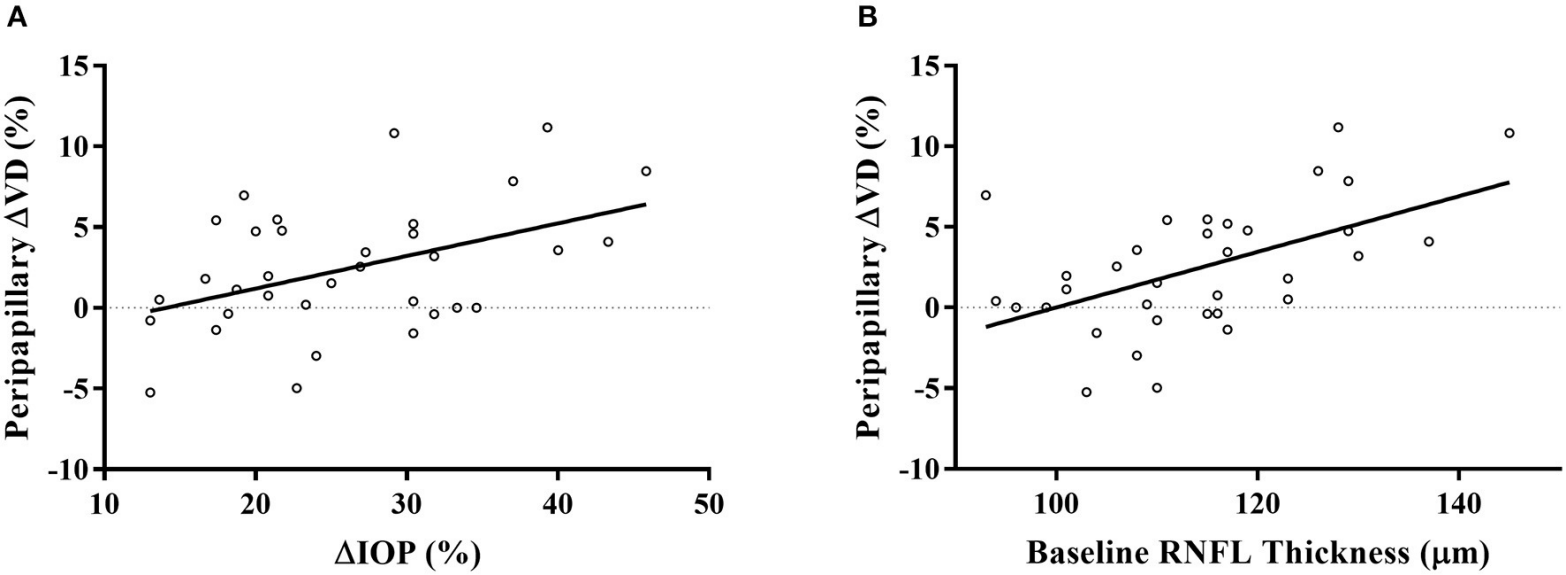
Scatterplots displayed the significant association of IOP reduction percentage **(A)** and baseline RNFL thickness **(B)** with the percentage of peripapillary VD increase after treatment. ΔVD represented the percentage of VD change after a 4-week latanoprost application. ΔIOP represented the percentage of IOP reduction of each subject.

As we found a significant increase in peripapillary RPC VD, we further explored its association with different parameters using linear regression analysis. Age, blood pressure, spherical equivalent, CCT, IOP, RNFL thickness, SQ difference between the visits, VD, and ONH measurements were included in the analysis. In the univariate model, age, percentage of IOP change, and the baseline RNFL thickness were significantly associated with the percentage of increase in peripapillary VD. Specifically, none of the other ONH parameters or their changes after IOP reduction was significantly correlated with peripapillary ΔVD. The further multivariate analysis displayed that both the percentage of IOP change (β = 0.330, *p* = 0.031) and baseline RNFL thickness (β = 0.450, *p* = 0.004) were significantly associated with the percentage of peripapillary VD improvement in the two models (Table 4; Figure 2).

**Table 4:** Factors associated with increase of peripapillary VD after treatment in linear regression analysis.

**Factors**	**Univariate model**			**Multivariate model**		
	**β**	**95% CI**	***p***	**β**	**95% CI**	***p***
Age	−0.525	(−0.252, −0.066)	**0.001**			
SBP	−0.052	(−0.181, 0.143)	0.810			
DBP	−0.139	(−0.213, 0.111)	0.519			
CCT	0.153	(−0.025, 0.062)	0.389			
SE	−0.157	(−0.759, 0.294)	0.375			
SQ difference	−1.015	(−2.639, 0.609)	0.212			
Baseline IOP	0.251	(−0.147, 0.902)	0.152			
Percentage of Δ IOP	0.447	(0.057, 0.347)	**0.008**	0.330	(0.015, 0.283)	**0.031**
Baseline VD	−0.108	(−0.722, 0.387)	0.543			
Baseline RNFL thickness	0.536	(0.075, 0.270)	**0.001**	0.450	(0.049, 0.240)	**0.004**
C/D area ratio	0.052	(−9.268, 12.426)	0.769			
C/D vertical ratio	0.008	(−7.602, 7.952)	0.964			
C/D horizontal ratio	0.007	(−8.545, 8.867)	0.970			
Rim area	0.120	(−2.636, 5.284)	0.501			
Disc area	0.105	(−2.093, 3.835)	0.554			
Cup volume	0.092	(−10.739, 18.101)	0.607			

*CCT, central corneal thickness; C/D, cup/disc; CI, confidence interval; DBP, diastolic blood pressure; IOP, intraocular pressure; RNFL, retinal nerve fiber layer; SBP, systolic blood pressure; SE, spherical equivalent; SQ, scan quality; VD, vessel density. P < 0.05 were marked in bold*.

## Discussion

The response of retinal microcirculation to IOP stimuli holds great promise to reveal the mechanism of the development and progression of glaucoma (28). Nonetheless, such relation was seldom reported on OHT patients (18, 23), who tend to progress into POAG with sustained high IOP (20, 21). The present study investigated the relationship of retinal VD and IOP change by latanoprost application using OCTA in OHT patients. To summarize, in OHT patients, (1) The peripapillary RPC VD increased significantly after lowering IOP. (2) The mild change of IOP did not alter the microcirculation in the macula. (3) Regression analysis indicated that the percentage of IOP change and the baseline RNFL thickness were both independent factors for the peripapillary RPC VD improvement.

The improvement of peripapillary RPC VD by long-term IOP reduction was shown on OHT patients. The OCTA data after the hypotensive treatment had proved a significant trend of increment in RPC VD (Table 2). Similar hypotensive treatment had shown little impact on ophthalmic artery blood supply (23). The conclusion was drawn based on the phase-contrast magnetic resonance imaging (PC-MRI) technique after the 1-week treatment of latanoprost (23). However, the applied technique's resolution was insufficient to assess the capillaries (29), which were autoregulated based on the local feedback and tissue demands (28). In comparison, OCTA displayed the structural signal of vessels and capillaries instead of functional signals like velocity (24, 25). It has shown good effectiveness in measuring VD of both children and adult OHT patients (18, 30). Currently, limiting studies have investigated the influence of medical anti-glaucomatous therapy on retinal microcirculation by OCTA. In a group of treatment-naïve subjects, latanoprost decreased the mean IOP by 26.1% but did not improve the peripapillary VD (18). However, due to the varying severity and subtypes of the including glaucoma patients, such a conclusion may be flawed. In our prospective study, we only enrolled OHT patients above the age of 10 years, with better compliance in IOP measurement (31). The peripapillary VD increased significantly with a mean IOP decrease of 26.3%. After Bonferroni adjustment, such difference became insignificant in all the sectors, but the increase of overall peripapillary VD was significant. Our study indicated the susceptibility to the IOP decrease was consistent among all the sectors. To further investigate the peripapillary microcirculation on glaucoma patients, previous studies had focused on surgical-induced IOP decrease (8–13). Within a 12-month follow-up period, filtering surgeries did not improve the peripapillary VD under an IOP reduction of 24.6–51.1% (8, 9, 11, 13). Specifically, Shin et al. observed improved peripapillary microcirculation in 61.3% of participants within three months, but the mean VD did not increase significantly for all the included patients (10). Such neutral response of ocular perfusion to anti-glaucomatous treatment may originate from the retinal vascular defects caused by glaucoma progression (28). Typically, those in need of glaucoma surgery may suffer advanced microcirculatory defects. In OHT patients, the regulatory function of peripapillary retinal vasculature was possibly preserved, which possibly explained our differences from previous studies. Our findings indicated that the RPC VD increased significantly after reducing IOP in OHT patients, revealing the potential of peripapillary vascular improvement after treatment.

The mild change of IOP did not alter the microcirculation in the macula. In our study, the IOP decreased by 6.5 ± 2.4 mmHg, much lower than the surgical-induced IOP decrease in the previous studies (8–11, 32). In OHT patients, unlike the peripapillary region, the macular VD measured in different layers and FAZ area remained stable (Table 3). Similar results were observed in glaucoma patients (8, 11, 13, 18). Within about one month, both the surgical (8, 11, 13) and medical (18) therapy failed to improve VD in the macula. Previous studies have revealed a more prominent vascular damage in the peripapillary areas (33). Compared with this region, macular VD had shown a lower diagnostic value for glaucoma (33–36). Therefore, the macular VD may be less susceptible to external factors. Despite this, with a more extended follow-up period after surgery, some claimed that the foveal VD had revealed an increasing trend (13), accompanied by a decreasing FAZ area (13, 32). It was postulated that such a pattern of VD change might be induced by surgical inflammation (13). However, such delayed trends were not observed in the peripapillary region, which was unparallel to its susceptibility. The exact role of surgical-induced IOP change on the retina still needs to be explored. As we had applied a topical medication to the participants, the induction of inflammation will be milder than surgery. During our one-month follow-up, the VD change of different layers in the foveal and parafoveal region revealed no significant difference, which further proved the insusceptibility of macular microcirculation toward IOP in OHT patients. Therefore, the mild change of IOP did not alter the vasculature condition in the macula.

The reduction of IOP and the baseline RNFL thickness were independent factors associated with peripapillary RPC VD improvement, as shown in the multivariate linear regression model (Figure 2; Table 4). Although the image quality (37) and myopic status (38) correlated with retinal VD, we did not find their correlation with VD improvement. Previous cross-sectional studies have demonstrated an insignificant prediction value of IOP on the measurements of peripapillary parameters (39, 40). To further explore the role of IOP change in the retinal microcirculation by OCTA, previous studies had provoked an IOP spike by laser peripheral iridotomy (LPI) (4, 7) or darkroom prone provocative test (3). Wang et al. had observed a significant correlation with RPC VD change and IOP spike after LPI, with a mean IOP increase of 6.3 mmHg (7). However, possibly due to the short duration of IOP increase, such correlation was insignificant in other studies, even with a more significant IOP reduction (3, 4). The existence of vascular autoregulation, which adjusts the retinal capillaries, may confound the response to acute pressure elevation (28). In our study, with a mild IOP change similar to Wang et al. (7), we observed a significant correlation after a long-term duration, indicating a prolonged influence of IOP. Similarly, the change of peripapillary VD was found to correlate with IOP change in glaucoma patients after three weeks (18), which adds robustness to our findings. Studies have shown abnormal ocular vascular autoregulation in glaucoma patients, indicating the reduction of vascular resistance to perfusion pressure changes (2). Given that the RPC is damaged with glaucoma progression (19), those in need of surgery may have impaired regulation patterns of retinal microcirculation compared to healthy controls or early-stage patients. It was reported that prostaglandin analog could improve retinal microcirculation (15, 17, 41), but the exact role of its interaction on IOP and microcirculation was not thoroughly studied. The pharmacological effect can't be completely ruled out unless further studies compare different doses or categories of IOP-lowering medication. Nevertheless, our results on OHT patients indicated the relationship between IOP and retinal blood supply on normal tissues. As for another factor, RNFL thickness remained stable after IOP reduction, but the baseline RNFL thickness correlated with peripapillary RPC VD improvement (Table 4). It was claimed that VD change introduced by IOP reduction after trabeculectomy in POAG was not associated with RNFL thickness (9). In normal structures, perfusion will be regulated based on the metabolic condition (2). However, as POAG patients have suffered RNFL and RPC defects (42), the relationship between RNFL thickness and RPC may be affected. Together, our study indicated that thicker RNFL might possess more significant potential to adjust the circulation conditions due to its metabolic requirement. The additional perfusion promoted by anti-glaucomatous treatments will increase the metabolic supply for peripapillary RNFL. Consequently, extra nourishment to the RNFL will be provided by IOP reduction, adding therapeutic value to the OHT patients. As topical hypotensive treatment had revealed its effectiveness in preventing or delaying POAG onset in OHT patients (20), the accompanying improvement of peripapillary microcirculation might serve as a protection mechanism. Together, both the IOP reduction and thicker baseline RNFL were independent factors for peripapillary RPC VD improvement.

Recent studies have focused on the application of OCT and OCTA on the early detection of development and progression in glaucoma suspects and patients. Specifically, the longitudinal structural and vascular metrics changes have been frequently studied (43, 44). In the early diagnosis of glaucoma, both ONH, RNFL, and macular parameters have shown a high level of value (44). For an experimental IOP spike, OCT detected an immediate change of vascular metrics (4, 7) and retinal structures (45, 46). While in varied surgical treatment for glaucoma patients, the OCT technique provides an objective index for follow-up (8–13, 47–50). Therefore, the current application of the OCT technique can provide information about the retinal response to stimulation or treatment for glaucoma patients. Similarly, our study shed light on the longitudinal influence of IOP lowering on the OHT, which indicated the potential of early treatment for these patients. Further studies comparing different medications or other IOP lowering techniques may be helpful in the early treatment of glaucoma.

There are still several limitations that need attention and further addressed in our study. First, we only included a relatively small sample of OHT patients whose retinal microcirculation may differ from glaucoma patients. The effect of latanoprost on the peripapillary microcirculation of glaucoma patients in different stages required further analysis. Second, the follow-up time of our study was limited to four weeks. The relationship between retinal microcirculation and IOP reduction induced by medications with a longer duration still needs to be explored, especially for treatment-naïve patients. Third, as measured by OCTA, vessel density did not display the exact velocity of retinal perfusion. Possible changes of velocity in both large vessels and microcirculation in the peripapillary region should be assessed in future studies.

In conclusion, the peripapillary VD in OHT patients increased after the reduction of IOP. The mild change of IOP did not alter the microcirculation in the macula. In addition, the percentage of IOP change and the baseline RNFL thickness were independent factors for the peripapillary RPC VD improvement.

## Data Availability Statement

The raw data supporting the conclusions of this article will be made available by the authors, without undue reservation.

## Ethics Statement

The studies involving human participants were reviewed and approved by Peking University Third Hospital. Written informed consent to participate in this study was provided by the participants' legal guardian/next of kin.

## Author Contributions

XC, YH, and CZ: conception and design. XC, YH, HD, QW, and DZ: data collection. XC, YH, and HD: analysis and interpretation of data. XC: drafting the manuscript. YH, HD, QW, DZ, and CZ: critical revesion of the manuscript. All authors contributed to the article and approved the submitted version.

## Funding

This study was supported by National Natural Science Foundation of China (No. 81970798) and Capital's Funds for Health Improvement and Research (No. CFH-2020-2-40911).

## Conflict of Interest

The authors declare that the research was conducted in the absence of any commercial or financial relationships that could be construed as a potential conflict of interest.

## Publisher's Note

All claims expressed in this article are solely those of the authors and do not necessarily represent those of their affiliated organizations, or those of the publisher, the editors and the reviewers. Any product that may be evaluated in this article, or claim that may be made by its manufacturer, is not guaranteed or endorsed by the publisher.
