# Supplementary material for: Change of Retinal Vessel Density After Lowering Intraocular Pressure in Ocular Hypertension
Source: Front Med (Lausanne). 2021 Dec 9;8:730327. doi: 10.3389/fmed.2021.730327 (PMC8695549; doi:10.3389/fmed.2021.730327)
Supplement: Supplementary file 1 [file Table_1.DOCX]

**Supplementary Table 1**

**Supplementary Table 1**: ICCs for OCTA VD and FAZ measurement in different regions.

| Sectors | ICC | 95%CI | *p* |
| --- | --- | --- | --- |
| Inside Disc | 0.993 | (0.974, 0.998) | **<0.001** |
| Peripapillary | 0.984 | (0.940, 0.996) | **<0.001** |
| Nasal Superior | 0.989 | (0.956, 0.997) | **<0.001** |
| Nasal Inferior | 0.975 | (0.901, 0.994) | **<0.001** |
| Inferior Nasal | 0.984 | (0.940, 0.996) | **<0.001** |
| Inferior Temporal | 0.996 | (0.984, 0.999) | **<0.001** |
| Temporal Inferior | 0.967 | (0.874, 0.992) | **<0.001** |
| Temporal Superior | 0.989 | (0.958, 0.997) | **<0.001** |
| Superior Temporal | 0.992 | (0.971, 0.998) | **<0.001** |
| Superior Nasal | 0.984 | (0.941, 0.996) | **<0.001** |
| Superficial Fovea | 0.999 | (0.994, 1.000) | **<0.001** |
| Superficial Parafovea | 1.000 | (0.998, 1.000) | **<0.001** |
| Temporal | 0.995 | (0.977, 0.999) | **<0.001** |
| Superior | 0.995 | (0.979, 0.999) | **<0.001** |
| Nasal | 0.976 | (0.846, 0.995) | **<0.001** |
| Inferior | 0.995 | (0.983, 0.999) | **<0.001** |
| Deep Fovea | 0.999 | (0.995, 1.000) | **<0.001** |
| Deep Parafovea | 0.999 | (0.996, 1.000) | **<0.001** |
| Temporal | 0.998 | (0.991, 0.999) | **<0.001** |
| Superior | 0.999 | (0.995, 1.000) | **<0.001** |
| Nasal | 0.998 | (0.992, 0.999) | **<0.001** |
| Inferior | 0.999 | (0.995, 1.000) | **<0.001** |
| FAZ | 1.000 | (0.999, 1.000) | **<0.001** |

*CI: confidence interval; FAZ: foveal avascular zone; ICC: intraclass correlation coefficient.
